# Attitudes towards Interprofessional education in the medical curriculum: a systematic review of the literature

**DOI:** 10.1186/s12909-020-02176-4

**Published:** 2020-08-06

**Authors:** Joana Berger-Estilita, Alexander Fuchs, Markus Hahn, Hsin Chiang, Robert Greif

**Affiliations:** 1 Department of Anaesthesiology and Pain Medicine, Inselspital, Bern University Hospital, University of Bern, Bern, Switzerland; 2 School of Medicine, Sigmund Freud University Vienna, Vienna, Austria

**Keywords:** Interprofessional education, IPE, Medical student, Pre-registration, Medical education, Attitudes, Medical curriculum

## Abstract

**Background:**

There is agreement among educators and professional bodies that interprofessional education needs to be implemented at the pre-registration level. We performed a systematic review assessing interprofessional learning interventions, measuring attitudes towards interprofessional education and involving pre-registration medical students across all years of medical education.

**Methods:**

A systematic literature review was performed using PubMed, PsycINFO, EThOS, EMBASE, PEDro and SCOPUS. Search terms were composed of interprofession*, interprofessional education, inter professional, inter professionally, IPE, and medical student. Inclusion criteria were 1) the use of a validated scale for assessment of attitudes towards IPE, and results for more than 35 medical students; 2) peer-reviewed articles in English and German, including medical students; and 3) results for IPE interventions published after the 2011 Interprofessional Education Collaborative (IPEC) report. We identified and screened 3995 articles. After elimination of duplicates or non-relevant topics, 278 articles remained as potentially relevant for full text assessment. We used a data extraction form including study designs, training methods, participant data, assessment measures, results, and medical year of participants for each study. A planned comprehensive meta-analysis was not possible.

**Results:**

This systematic review included 23 articles with a pre-test-post-test design. Interventions varied in their type and topic. Duration of interventions varied from 25 min to 6 months, and interprofessional groups ranged from 2 to 25 students. Nine studies (39%) reported data from first-year medical students, five (22%) from second-year students, six (26%) from third-year students, two (9%) from fourth-year students and one (4%) from sixth-year students. There were no studies including fifth-year students. The most frequently used assessment method was the Readiness for Interprofessional Learning Scale (RIPLS) (*n* = 6, 26%). About half of study outcomes showed a significant increase in positive attitudes towards interprofessional education after interventions across all medical years.

**Conclusions:**

This systematic review showed some evidence of a post-intervention change of attitudes towards IPE across different medical years studied. IPE was successfully introduced both in pre-clinical and clinical years of the medical curriculum. With respect to changes in attitudes to IPE, we could not demonstrate a difference between interventions delivered in early and later years of the curriculum.

**Trial registration:**

PROSPERO registration number: CRD42020160964.

## Background

According to the World Health Organization (WHO), Interprofessional Education (IPE) occurs when “students from two or more professions learn about, from, and with each other to enable effective collaboration and improve health outcomes” [1]. Safe, high-quality, accessible, patient-centred care requires continuous development of interprofessional competencies [2], and IPE has repeatedly been called for, so that healthcare students can enter the workforce as effective collaborators [3–5].

A growing amount of empirical work shows that IPE can have a beneficial impact on learners’ attitudes, knowledge, skills, and behaviours (the so-called collaborative competencies) [6, 7], and can positively affect professional practice and patient outcomes [8, 9]. IPE may enhance attitudes toward teamwork and collaboration, leading to improved patient care upon graduation. However, the optimal time to expose medical students to IPE is still subject to debate.

IPE may enhance attitudes toward collaboration and teamwork during training, leading to improved attitudes towards IP upon graduation. Nevertheless, the complexity of simultaneous teaching for different healthcare disciplines, as well as logistical problems and busy timetables raise issues concerning the introduction of IPE interventions. The optimal timing to introduce IPE and whether immersion (i.e. continuous collaborative learning) or exposure (periodic collaborative activities) should be adopted [10] are still subject to debate. Gilbert [11] suggests exposure during the early years and immersion in the graduation year. Reasons for this include ensuring the optimal development of students’ professional identity before expecting them to work collaboratively with others. Furthermore, delaying the introduction of IPE to later in the curriculum may be deterred by the students’ focus on profession-specific clinical practice, and immersion in vocation-specific stereotypes or negative attitudes [10]. Current undergraduate literature shows a tendency to introduce IPE earlier, even in the first year of studies [11, 12], but the most effective timing to perform PE interventions in the medical curriculum remains to be determined.

We undertook a systematic literature review to determine the most effective time to introduce IPE to pre-registration medical students. Additionally, we were interested in exploring the nature of the training, the assessment methods and the study outcomes. Our systematic review was guided by the research question: “What is the optimal time to institute interprofessional education interventions in the medical school curriculum?”

## Methods

### Study design

We performed a systematic review of the literature focusing on interprofessional learning interventions in pre-registration medical students and applied a review protocol based on the PRISMA (Preferred Reporting Items for Systematic Reviews and Meta-analyses) statement [13]. We also aimed to perform a meta-analysis with studies grouped by type of assessment. This systematic review was registered in PROSPERO (www.crd.york.ac.uk) with the number CRD42020160964.

### Data sources and selection criteria

The systematic literature search was performed on December 12, 2019, using the databases PubMed, PsycINFO, EThOS, EMBASE, PEDro and SCOPUS. The following keywords and subject headings were used as search terms: interprofession*, *interprofessional education*, *inter professional*, *inter professionally*, *IPE*, and *medical student*. We included all peer-reviewed articles in English and German that reported on evaluative studies of IPE interventions including medical students, and were published after the 2011 Interprofessional Education Collaborative (IPEC) report [2]. The full search strategy is available in an additional word file [see Additional file 1]. In addition, we included articles found in the reference lists of previous reviews on IPE, discovered as a result of the search for IPE interventions [4, 6, 9, 14–22].

### Inclusion criteria

We included studies that reported on assessment of knowledge, skills or attitudes (KSA), with an IPE intervention, and that reported quantitative results with a validated IPE instrument. We included only studies using previously comprehensive validated instruments according to various psychometric tests. Validated questionnaires provide reliable and valid results, and can be used to benchmark or compare results on an international level [23], and make statistical comparisons, therefore increasing rigour and allowing for a meta-analysis. One limitation of the use of validated questionnaires is the lack of further piloting or cultural adaptation, which may induce bias. We also narrowed our search to groups of at least 35 medical students in the same year of their medical education programme, to ensure an adequate sample size for statistical validity. To avoid interventions in overlapping years of education, we selected studies reporting on interventions with a duration of at most 6 months (regardless of the type of intervention, the study programme, and the educational year of other students taking part). Although we encountered qualitative IPE studies, we chose a positivist approach because it better aligned with our intention to perform a meta-analysis.

### Exclusion criteria

We excluded conference contributions and abstracts without a related peer-reviewed published article. We also excluded all non-validated questionnaires and articles without available full-text in English or German.

### Identification of potentially eligible studies

**Fig. 1:**
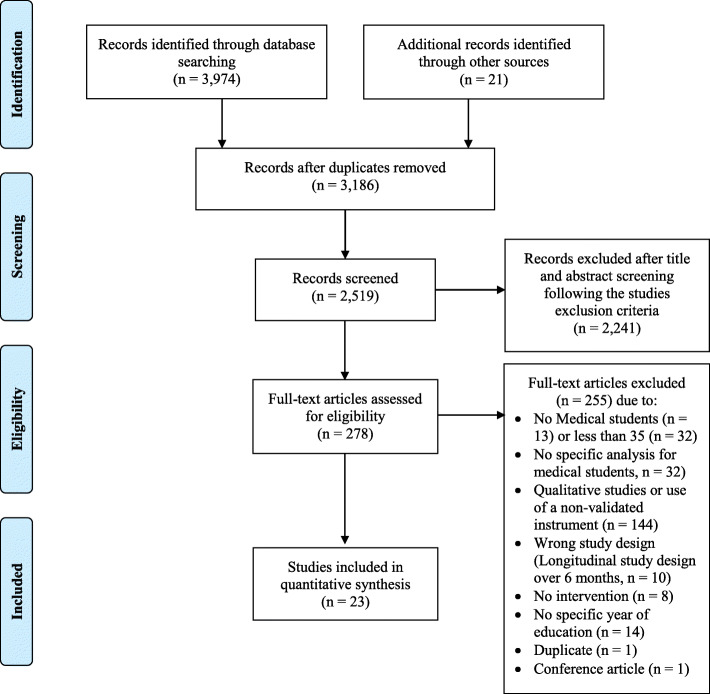
PRISMA Study Flow diagram

After the primary search, all titles and abstracts were screened and duplicates or non-relevant articles were excluded. The full text of the remaining articles was read by two authors (JBE and AF) to identify the eligible articles for this review. All potentially eligible articles were imported into a software platform for systematic reviews (http://rayyan.qcri.org) [24] to expedite the screening of abstracts and titles and to determine the final selection of eligible studies. The two authors initially performed selection in a blinded mode with three options: “include”, “exclude” and “maybe”. After finishing the first personal assessment, results were unblinded and disagreements were resolved by discussion of individual papers to find consensus. The study selection process is outlined in the PRISMA Flow Diagram – Fig. 1.

### Data extraction and synthesis

The data extraction form was developed by two reviewers, informed by the form from Reeves et al. [9] but modified to include important aspects specific to this review, including ratio of study year to total duration of studies and classification of “early” or “late” depending if the IPE intervention occurred in the first or second half of medical studies. The reviewers extracted additional data regarding the context of study, recruitment, description of participants, study design, results and conclusions. The analysis of the risk of bias was performed independently, at a later stage. RG moderated in case of disagreement.

Upon completion of article extraction, data were analysed using the Statistical Package for the Social Sciences (SPSS). 23.0. (IBM Corp., Armonk NY, USA). We report descriptive statistics for quantitative data (median, IQR). Data extracted were synthesised in a narrative manner, using an integrative and aggregative approach [25].

### Quality assessment and risk of bias

The quality of included studies was also evaluated by JBE and HC using a standardised critical appraisal tool, the McMaster Critical Review Form for Quantitative Studies [26]. If research articles met each criterion outlined in the appraisal guidelines, they received a score of “one” for that item, or, if they did not, a score of “zero”. Item scores were then summed to provide a score of a maximum of 16, with 16 indicating excellent methodological rigour. The quality was defined as poor when the overall score was 8 or less, fair if 9–10, good if 11–12, very good if 13–14 and excellent if 15–16 [27]. This tool was chosen for this systematic review as it is published, freely available, has been used extensively, and can be applied to a range of research designs [28]. Differences in judgment were resolved through discussion.

### Statistics

A meta-analysis for those studies using the Readiness of Interprofessional Learning Scale (RIPLS) [10, 29–33] was attempted with the R meta package [34], as this scale was most often used. Otherwise, descriptive analyses were conducted, including frequencies. Where applicable, scales were reversed by subtracting the mean from the maximum score for the scale to ensure a consistent direction of effects across studies. Weighted means of subscales were calculated for each study using the number of participants as weights. Pooling of estimates on the single-item level was not possible, as Sheu et al. [30] only reported on subscale level. Estimates of weighted means of subscales are reported with 95% confidence intervals (CIs). A random effects model was used with the inverse variance method for pooling of estimates across the remaining studies using RIPLS. Standard deviations of mean changes were not given and had to be calculated according to Cochrane’s Handbook [35], which introduced further uncertainty by the need to choose a more or less random correlation coefficient for standard deviations.The meta-analysis was conducted using R 3.5.0 statistical package (R Foundation for Statistical Computing, Vienna, Austria) after related content was extracted and all remaining analyses were conducted by SPSS v.23 (IBM Corp. in Armonk, NY, USA).

## Results

### Trial flow

The literature search retrieved 3995 articles. After applying the inclusion and exclusion criteria and removing duplicates, 23 articles were included in the review [10, 29–33, 36–52] (see PRISMA Flow diagram, Fig. 1). All studies had a pre-test-post-test design. Basic characteristics of educational interventions are presented in Table 1. We present an overview of characteristics of the included studies in Table 2.

**Table 1:** Categorised description and characteristics of the 23 included studies (Findings of individual studies could belong to more than one category)

Category	n (%)
**Study design**	
*cross-sectional*	16 (64)
*prospective cohort*	2 (8)
*quasi-experimental*	4 (16)
*randomised*	2 (8)
*mixed-methods*	1 (4)
with pre-test-post-test assessment	23 (100)
**Frequency of course**	
*single time activity*	11 (47.8)
*multiple times occurring during the year*	2 (8.7)
*annually*	10 (43.5)
**Duration of educational intervention**	
*< 6 h*	9 (39.1)
*> 6 h, < 1 week*	2 (8.7)
*1–8 weeks*	7 (30.4)
*over 8 weeks, up to one semester*	5 (21.7)
**Educational strategies (*n*** **= 44)**	
*small-group discussion*	7 (15.9)
*simulation*	6 (13.6)
*workshops*	5 (11.4)
*large-group lecture*	4 (9.1)
*community-based projects*	4 (9.1)
*reflective exercises*	4 (9.1)
*clinical teaching/direct patient interaction*	3 (6.8)
*patient case analysis*	2 (4.5)
*shadowing*	2 (4.5)
*eLearning*	2 (4.5)
*other (*e.g.*, family visits, joint lab sessions)*	5 (11.4)
**Professions represented**	
*only medical students*	3 (13)
two*	12 (52.2)
three*	4 (17.4)
four professions or more*	4 (17.4)
**Outcomes (*n*** **= 49)**	
*attitudes*	38 (77.6)
*satisfaction*	8 (16.3)
*skills*	1 (2)
*other*	2 (4.1)
***Assessment methods (n = 46)***	
self-reported questionnaire *(attitudes/satisfaction)*	35 (76.1)
*debriefs/interviews/focus groups*	1 (2.2)
*program feedback/evaluation*	4 (8.7)
*knowledge test*	1 (2.2)
*ratings for skill performance*	2 (4.3)
*other*	3 (6.5)
Reliability reported	4 (12.8)
Validity reported	4 (12.8)

**Table 2:** Extraction grid for selected studies

Study	Design	Country	Year*	Educational intervention	Research objectives	Duration	Type & number of students	Group size	Name & number of outcomes	Results
Chua et al. [28]	pre-test-post-test	SGP	1/5 Early	Student Medical Education Conference 2013 (IPE components) with IP workshops and plenary sessions	Effectiveness of an IP conference in improving attitudes towards IPE	N/A	MS (*n* = 281; 79,8%) NS (*n* = 71;20,2%) total *n* = 352	N/A	RIPLS *n* = 1	**Significantly increase** in post-test; pre-test M (SD) = 81.54(7.36) vs post-test M (SD) = 85.51(8.08); (***p < 0.001)*** Students with previous IPE experience scored higher in the pre-test
Hawkes et al. [29]	pre-test-post-test	UK	1/5 Early	7-week IPE preparation of a care management plan	Assessment of 1st-year students’ attitudes towards other HCP	7 weeks	MS (*n* = 100; 45,2%) PS (*n* = 68; 30,8%) NS (*n* = 53; 24%) total *n* = 221	N/A	AHPQ *n* = 1	All professions saw a statistically **significant increase** (**p < 0.01**) in how ‘**caring**’ they were perceived to be by all students after IPL
Hess et al. [30]	pre-test-post-test	USA	1/4 Early	Communication skills: asynchronous, online, self-directed learning modules, alternating with live small group session; Recording of SP	Effectiveness of a course in teaching patient-centred IP communication	4 × 20 hours in 6 months	MS (*n* = 67; 54%) PS (*n* = 57; 46%) total *n* = 124	6–7	CGI *n* = 1	Communication skill construct scores & global rating scores (pre-test M (SD) = 2.2(0.5); Median (IQR) = 2.0(2.0–2.5) vs post-test M (SD) = 4.1(0.6); Median (IQR) = 4.0(3.8–4.5) **significantly increased** for both (MS&PS) post-test compared to pre-test (***p*** **< 0.01**) pre-test scores for rapport building higher for MS
Hudson et al. [11]	Cross-sectional pre-test-post-test	AUS	1/4 Early	Chronic care; ICE with local HCP teams	Exploration of changes in MS attitudes toward IPL & patient-centred care	3 weeks in total	MS (*n* = 279; 100%) total *n* = 279	N/A	RIPLS *n* = 1	post-test scores **significantly decreased**; negative attitudes towards IPE: teamwork and collaboration (M = 40.64, SEM = 0.21 vs M = 39.45, SEM = 0.25; p < 0.001); professional identity (M = 30.53, SEM = 0.21 vs M = 29.18, SEM = 0.23; *p* < 0.001); patient-centredness (M = 23.42, SEM = 0.11 vs M = 23.07, SEM = 0.13; *p* < 0.01)
Quesnelle et al. [31]	pre-test-post-test	USA	1/4 Early	Medication; Multi-institutional tele-health TBL event on pharmacogenomics; unique treatment plan for a patient	Assessment of a multi-institutional tele-health TBL activity	2 h (SS)	MS (*n* = 67; 74,4%) PS (*n* = 23; 25,6%) total *n* = 90	8	SATP2C *n* = 1	Post-test **significantly increased** Results for shift by category: Responsibility and Accountability M (SEM) = 0.21(+/−.06), p < 0.005; Shared Authority M (SEM) = 0.08(+/−.06), *p* < 0.005; Interprofessional Education M (SEM) = 0.14(+/.05), *p* < 0.05; Pharmacogenomics Confidence M (SEM) = 0.56(+/−.09), *p* < 0.005
Sheu et al. [32]	prospective cohort study, pre-test-post-test	USA	1/4 Early	Student-Run Clinic, including preparatory didactic sessions on health disparities & cultural competencies appropriate to the target population & work in the clinic	Analyse the impact of student-run clinic on students	Variable	MS (*n* = 93; 51,1%) NS (*n* = 31; 17%) PS (*n* = 58; 31,9%) total *n* = 182	N/A	SAMI RIPLS *n* = 2	**No changes for MS but previous high positive attitudes** RIPLS M (SD) pre vs post by subscales: Team: 4.49 (0.44) vs 4.32 (0.44); Identity: 4.31(0.51) vs 4.04(0.56); Role: 2.71(0.61) vs 2.85(0.68) SAMI M (SD) pre vs post: Exposure 4.02 (.58) vs 4.03 (.58); Perception 3.76(.70) vs 3.83(.67)
Sytsma et al. [33]	quasi-experimental pre-test-post-test	USA	1/4 Early	mono and IP teams interventions: 1. Social event, 2. Peer-teaching in Anatomy dissection lab, and 3. Collaborative clinical problem-solving sessions	Describe an IPE experience in gross anatomy and report its lasting impact	7 weeks	MS (*n* = 35; 66%) PT (*n* = 18; 34%) total *n* = 53	8–12	RIPLS *n* = 1	Overall, students showed **positive attitudes towards IPE, no significant changes** in the post-test; pre vs post total M (SD): 81.54(7.36) vs 85.51(8.08);
Tuirán-Gutiérrez et al. [34]	experimental randomised pre-test-post-test	SP	1/7 Early	Experimental group received IP training in collaborative work (control group received training on drug addiction prevention)	Are there role differences in early IP training? Does IP training improve attitudes towards IPE?	18 × 2 hours over 4 months	MS (*n* = 84; 48,6%) NS (*n* = 89; 51,4%) total *n* = 173	N/A	JSE JSAPNC JeffSPLL *n* = 3	Post-test scores for MS in the IPE group remained stable Pre-vs post M (SD) JSAPNC: 48(6) vs 48(7); JSE-S: 104(12) vs 100(14) JeffSPLL-MS: 46(4) vs 46(6) **significant deterioration in the control group** in the development of collaborative work skills, pre vs post control group: M (SD) = 47(6) vs 44(7) *p* < 0.01
Van Winkle et al. [35]	randomized, prospective cohort study pre-test-post-test	USA	1/4 Early	IP Workshop: Session 1: Management of 2 cases Session 2: individual reflection exercise	Measure changes in collaboration scores after an IP workshop	2 × 50 min (2–7 days)	PS (*n* = 215; 42,8%) MS (*n* = 205; 40,8%) BM (*n* = 82; 16,3%) total *n* = 502	6	SATP2C Modified JSE *n* = 2	High baseline commitment scores did not change for MS: SATP2C max 65: pre M = 54; education component increased significantly after IPE for 82% of MS (*p* = 0.015), the increase in scores for the other 2 factors (13 items) was not significant (*p* = 0.76) modified JSE correlated positively (Pearson correlation coefficient (r) values was 0.38 (p,0.0001)
Haber et al. [36]	pre-test-post-test	USA	2/4 Early	IP simulation: SP for physical examination providing patient-centred care of an older adult with diabetes and periodontal disease	Effectiveness of an IP clinical simulation and case study experience	1 h (SS)	MS (*n* = 310; 50,2%) NS (*n* = 150; 24,3%) DS (*n* = 158; 25,6%) total *n* = 618	8	ICCAS *n* = 1	Significant change (*p* < 0.001) in ICCAS and each of the 6 IP-competency domains (*p* < 0.0001) pre vs post M 4.63 vs 5.3/5.4 MS had lower mean post-test scores compared to other students
McCaffrey et al. [37]	interventional study, pre-test-post-test	USA	2/3,5 Late	IP team approach: diagnosis and treatment of dementia with (1) informative session and (2) participation in five clinical exercises	Enhanced competency in Alzheimer’s & IP approach to roles of care	15 weeks	MS (*n* = 74; 61,7%) NS (*n* = 46; 38,3%) total *n* = 120	2	ATITS ATCS *n* = 2	MS with higher initial scores on knowledge test; **significant increase** in positive attitudes toward patients, disease and opinion post-test in the intervention group compared to control group (*p* = 0.02); attitudes towards IPE remains stable
Pinto et al. [38]	pre-test-post-test	USA	2/4 Early	Simulation (stroke, assessing the patient & developing a care plan, followed by debriefing)	Examination of an IP stroke simulation with SP on student IP growth	50 min	MS (*n* = 70; 37,2%) PA (*n* = 12; 6,4%) NS (=44; 23,4%) PT (*n* = 28;14,9%) OT (*n* = 34; 18,1%) total *n* = 188	5	IPEC *n* = 1	MS with **significant increase** in “values” and “interaction” post-test: IPEC: Values Domain: Mean Diff 0.79; 95% CI 0.13–1.45; *p*-Value 0.0205; Interactions Domain: Mean Diff: 1.91; 95% CI 1.07–2.76; p-value < 0.0001
Shrader et al. [39]	randomized pre-test-post-test	USA	2/4 Early	MUSC Senior Mentor Program: in-home interview, medication history, identification of medication-related issues & group discussions	Impact of a geriatric medication activity on student’s attitudes towards IPE and determination of student satisfaction	12 h/ Semester	MS (n = 101; 64,7%) PS (*n* = 55; 35,3%) total *n* = 156	3	SATP2C *n* = 1	Post: two items significant increased, one item significant decrease in attitudes for MS
Zanotti et al. [40]	pre-test-post-test	IT	2/6 Early	Interactions with HCP with (1) on-site observation and (2) review of experience in IP activities	Improved attitudes towards IP teamwork in MS after a new program	50 h over 1–2 weeks	MS (*n* = 277; 100%) total *n* = 277	N/A	IEPS CSI *n* = 1	**Significant** **improvements** after IPE training in 3 items (mem) and all item (women) M (SD) pre vs post: Competency & Autonomy 29.23 (3.51) vs 31.17 (3.58); ***p < 0.001***; Perceived need for cooperation 8.52 (1.21) vs 8.89 (1.12); ***p*** **< 0.001**; Perception of actual cooperation 17.67 (2.92) vs 19.43 (2.90); **p < 0.001**; Understanding others’ values 9.99 (1.62) vs10.30 (1.65); ***p*** **= 0.005** (only for women significant)
Berger et al. [41]	intervention, comparison group, pre-test-post-test	GER	3/6 Early	team communication seminar (eMonoprofessional MP (MS) compared with IP small groups)	Develop, “pilot” and evaluate a seminar on team communication	3½ hours (SS)	MS (*n* = 145; 87,9%) NS (*n* = 20; 12,1%) total *n* = 165	10–12	UWE-IP-D *n* = 1	**Significant positive changes** Communication and Teamwork Scale (Pre: M (IP) = 18.5;M (MP) = 18.0; *p* = 0.82) and post both showed significant (p < 0.01) positive changes (Post: M (IP) = 17.2, M (MP) = 17.4); Interprofessional Learning Scale IP group more positive baseline mean score (Pre: M (IP) = 20.6, M (MP) = 25.8; *p* < 0.01). both groups showed significant (*p* < 0.01) positive changes (Post: M (IP) = 19.1, M (MP) = 23.3)
Bridgeman et al. [42]	pre-test-post-test	USA	3/4 Late	IPE workshop “medication errors prevention”	Expose learners to IPE competencies and compare pre- to post workshop changes	3 h (SS)	MS (*n* = 43; 21,4%) PS (*n* = 140; 69,7%) PA (*n* = 18; 8,9%) total *n* = 201	5	ATHCTS *n* = 1	Attitudes improved after IPE, although MS attitudes improved only for team values (subscale 1): Subscale 1 pre % max core vs post % max score 73.0 ± 12.8 vs 76.9 ± 15.8 95% CI − 3.93 (− 6.59, − 1.27), *p* = 0.005; subscale 2 pre % max core vs post % max score 73.3 ± 13.7 vs 75.0 ± 17.2; 95 CI − 1.67 (− 4.74, 1.39); *p*-value n.s.; subscale 3 pre % max core vs post % max score 42.6 ± 17.0 vs 46.4 ± 12.5; 95% CI − 3.84 (− 8.43, 0.75); p-value n.s.
Friman et al. [43]	Mixed-methods exploratory, pre-test-post-test	SWE	3/6 Early	IPE workshop skill stations (Doppler assessment & compression therapy) + 1 case-based reflection on professional identity	Influence of a shared learning activity on attitudes towards IPE	3 h (SS)	MS (*n* = 101; 45,7%) NS (*n* = 120; 54,3%) total *n* = 221	2	JSAPNC *n* = 1	No differences in the MS group over time but initial high scores: pre vs post sum score means (max 60): 51.76 vs 51.76
Erickson et al. [44]	pre-test-post-test	USA	3/4 Late	IPE workshop “Difficult Discussions” (EOL care & communication, simulation)	Reports outcomes after IPE workshop	1½ hours (SS)	MS (*n* = 71; 53%) NS (*n* = 63; 47%) total *n* = 134	25	JSAPNC ATHCTS SEIEL *n* = 3	MS had higher scores post IPE in the ATHCT post-intervention (Mean (SD) 2.1 (6.1); *p* = .004 and “Quality Process in Teams” Mean (SD) 2.2 (5.6) *p* = .001, no change on the total scale
Oza et al. [45]	Cross-sectional observational pre-test-post-test	USA	3/4 Late	OSCE: Interprofessional case	Relationship between attitudes towards IPE & (1) self-efficacy, (2) prior extracurricular IP, (3) previous IPE	25 min	MS (*n* = 464; 100%) total *n* = 464	N/A	SEIEL *n* = 1	Students’ self-efficacy for IP was associated with IP collaborative practice, self-efficacy for feedback and evaluation were not; Mean SEIEL scores were high. For factor 1, interprofessional teamwork, M (SD) 7.9 (1.3, range 2.0–10.0) and for factor 2, interprofessional feedback and evaluation 7.1 (1.5, range 1.3–10.0).
Paige et al. [46]	quasi-experimental pre-test-post-test	USA	3/4 Late	Acute care; Simulation (dual major trauma scenarios with immediate structured debriefing)	Does IP SBT change behaviour over the course & is it as effective as team training?	2 h (SS)	MS (*n* = 118; 47,8%), NS (*n* = 129; 52,2%) total *n* = 247	3–8	TAS RIPLS T-TAQ *n* = 3	**Statistically significant improvement** in 10 of 19 RIPLS items, particularly in teamwork and team-based skills, T-TAQ: statistically significant improvements in the team structure subscale
Darlow et al. [47]	prospective controlled trial, pre-test-post-test	NZ	4/6 Late	Chronic care, IP workshops: people with LTC, e-learning platform, visits to a patient in the community, peer-presentation, group discussion	Evaluate if an IPE programme for managing people with LTC changes students’ attitudes to IP teams	11 h over 4 weeks	MS (*n* = 36; 43,4%), PT (*n* = 12; 14,5%) RT (*n* = 26; 31,3%) DiS (n = 9; 10,8%) total *n* = 83	3	ATHCTS RIPLS TSS *n* = 3	Mean post-intervention attitude scores were **significantly higher in the intervention group** than the control group for all scales. The mean difference for the ATHCTS was 0.17 (95%CI 0.05 to 0.30; *p* = 0.006); RIPLS was 0.30 (95%CI 0.16 to 0.43; *p* < 0.001), TSS was 0.71 (95%CI 0.49 to 0.92; *p* < 0.001)
Lockeman et al. [48]	quasi-experimental pre-test-post-test	USA	4/4 Late	Acute care, simulation: collaboration around acutely ill patients (ACLS algorithms)	Can a series of IP SBT promote changes in attitudes & stereotypes of HCP students	3x2hours over 2 weeks	MS (*n* = 163; 51,1%), NS (*n* = 156; 48,9%) total *n* = 319	6–7	SPICE-R2 HSS *n* = 2	No changes in HSS for MS **Statistically significant increase** in SPICE-R2 ratings from pre- to posttest: SPICE-R2 total scale pre M (SD) vs post M (SD) 4.23(0.47) vs 4.56(0.42), p < 0.001; Teamwork subscale pre vs post 4.18(0.52) vs 4.55(0.46), p < 0.001; Roles/Responsibilities subscale 4.00(0.58) vs 4.41(0.50), p < 0.001; Patient Outcomes subscale 4.52(0.48) vs 4.71(0.41), p < 0.001;
Seaman et al. [49]	descriptive matched before-after study, pre-test-post-test	AUS	6/6 Late	Ambulatory clinical placement, two of four clinical outpatient areas, in IP pairs, interact with HCP supporting the care of patients with chronic illnesses in hospital outpatient clinics and during home visits	Examine students’ beliefs, behaviours and attitudes in relation to IP socialisation in ambulatory care	2 weeks	MS (*n* = 45; 72,6%) NS (*n* = 17; 27,4%) total *n* = 62	2	ISVS *n* = 1	Significant increase of ISVS score in posttest with a mean improvement of 6.76 for the overall ISVS score (p < 0.001)

*ratio of study year to total duration of studies and classification of “Early” or “Late” depending if the IPE intervention occurred in the first or second half of medical studies

Abbreviations: countries: *SGP* Singapore, *UK* United Kingdom, *USA* United States of America, *AUS* Australia, *SP* Spain, *IT* Italy, *GER* Germany, *SWE* Sweden, *NZ* New Zealand; interventions: *IPE* interprofessional education, *IP* interprofessional, *IPL* interprofessional learning, *MP* monoprofessional; *TBL* team-based learning =, *HCP* health care professional, *SP* standardised patient, *ICE* interdisciplinary clinical experience, *SBT* simulation-based training, *OSCE* objective structured clinical examination, *ACLS* advanced cardiac life support, *LTC* long-term conditions, *EOL* end-of-life; students: *MS* medical, *NS* nursing, *PS* pharmacy, *PT* physical therapy, *BM* biomedical science, *DS* dental medicine, *RT* radiation therapy, *DiS* dietetics, *PA* physician’s assistant, *OT* occupational therapy; instruments: *RIPLS* Readiness for Interprofessional Learning Scale, *AHPQ* Attitudes to Health Professionals Questionnaire, *CGI* Common Ground Instrument, *SATP2C* Scale of Attitudes toward Physician-Pharmacist Collaboration, *SAMI* Sociocultural Attitudes in Medicine Inventory, *JSE* Jefferson Scale of Empathy, *JSAPNC* Jefferson Scale of Attitudes toward Physician-Nurse Collaboration, *JeffSPLL* Jefferson Scale of Physician Lifelong Learning, *ICCAS* Interprofessional Collaborative Competency Attainment Scale, *ATCS* Attitudes Towards Collaboration Scale, *ATITS* Attitudes Toward Interdisciplinary Teams Scale, *IPEC CSI* Interprofessional Education Collaborative IPEC Competency Self-assessment Instrument, *IEPS* Interdisciplinary Education Perception Scale, *UWE-IP-D* University of the West of England Interprofessional Questionnaire (German Version), *ATHCTS* Attitudes Towards Health Care Teams Scale, *SEIEL* Self-Efficacy for Interprofessional Experimental Learning, *TAS* Teamwork Assessment Scale, *T-TAQ* Team Strategies and Tools to Enhance Performance and Patient Safety (TeamSTEPPS) Teamwork Attitude Questionnaire, *TSS* Team Skills Scale, *SPICE-R2* Student Perceptions of Interprofessional Clinical Education, *HSS* Healthcare Stereotypes Scale, *ISVS* Interprofessional Socialization and Valuing Scale; results: *M* Mean, *SD* Standard deviation, *vs* versus, *SEM* standard error of the mean, *IQR* interquartile range, *p p*-value, 95%; *CI* confidence intervall

### Participants

In total 5231 students, of which 62% (*n* = 3229) were medical students, experienced an IPE intervention. The median number of MS in the IPE interventions was 100 [35–464]. Nine studies (39%) reported data for first-year medical students [10, 29–31, 36–40], five (22%) for second-year students [41–45], six (26%) for third-year students [32, 46–50], two (9%) for fourth-year [33, 51] and one for sixth-year medical students [52]. No study reported interventions occurring in the fifth year. Most studies (65%) [10, 29–31, 36–41, 43–46, 48] were performed in the first half of the medical curriculum. Three studies [10, 45, 50] (13%) involved only medical students. In all the interventions across all the studies, the other professional groups in the IPE intervention included nursing, pharmacy, dental medicine, physical therapy, biomedical science, occupational therapy, physician’s assistant, radiotherapy and dietetics students (Table 2).

### Study designs and locations

The study design was mainly cross-sectional (*n* = 16). Only two studies (9%) were randomised [39, 40]. Most studies took place in the USA (*n* = 14) [30–32, 37, 38, 40–44, 47, 49–52] and in Europe (*n* = 5, Germany, Italy, Spain, Sweden and the United Kingdom) [36, 39, 45, 46, 48].

### Interventions

Interventions varied in their type and topic. Most frequently, faculty chose IPE interventions on the topic of chronic care [e.g., Alzheimer’s disease [42], end-of-life issues [49], geriatric care [44], long-term conditions [10, 33, 36, 41, 52] (*n* = 8)] or acute care (*n* = 4) [30, 32, 43, 51]. Other topics were communication (*n* = 2) [37, 46]; medication plans and errors (*n* = 3) [38, 44, 47] and teaching aimed at influencing interprofessional knowledge, attitudes and skills [29, 31, 39, 40, 45, 48, 53]. Duration of interventions varied from 25 min [50] to 6 months [37], and interprofessional group size ranged from 2 [42, 48] to 25 [49] students. The main educational strategies were small group discussions (*n* = 7) [30, 31, 36–38, 47, 48], simulations (*n* = 6) [32, 41, 43, 49–51] and workshops (*n* = 5) [38–40, 44, 47]. The majority of the reported interventions (48%, *n* = 11) were held a single time, and 39% (*n* = 9) lasted less than 6 h.

### Assessment measures and outcomes

All studies reported learning outcomes. We could identify 49 different outcome measurements with 46 different assessment methods, but the majority (76%, *n* = 35) were questionnaires. The most frequent outcomes were attitudes towards IPE and/or other professions (78%, *n* = 38) and satisfaction (16%, *n* = 8). Eight studies (35%) used more than one validated instrument to evaluate the experience; four studies [30, 40, 42, 51] used two instruments, and the other four [32, 33, 39, 49] used three. The most commonly used method for assessing attitudes towards IPE was the RIPLS, used in six studies (26%) [10, 29–33], but a total of 22 different scales were used:

- Attitudes to Health Professionals Questionnaire (AHPQ) [36]
- Common Ground Instrument (CGI) [36]
- Scale of Attitudes toward Physician-Pharmacist Collaboration (SATP2C) [38, 40, 44]
- Sociocultural Attitudes in Medicine Inventory (SAMI) [30]
- Jefferson Scale of Empathy (JSE) [39, 40]
- Jefferson Scale of Attitudes toward Physician-Nurse Collaboration (JSAPNC) [39, 48, 49]
- Jefferson Scale of Physician Lifelong Learning (JeffSPLL) [39]
- Interprofessional Collaborative Competency Attainment Scale (ICCAS) [41]
- Attitudes Toward Collaboration Scale (ATCS) [42],
- Attitudes Toward Interdisciplinary Teams Scale (ATITS) [42]
- Interprofessional Educative Collaborative Competency Self-Assessment Instrument (IPEC CSI) [43]
- Interdisciplinary Education Perception Scale (IEPS) [45]
- University of the West of England Interprofessional Questionnaire (UWE-IP-D) [46]
- Attitudes Towards Health Care Teams Scale (ATHCTS) [33, 42, 47, 49]
- Self-Efficacy for Interprofessional Experimental Learning (SEIEL) [50]
- Teamwork Assessment Scale (TAS) [32]
- Team Strategies and Tools to Enhance Performance and Patient Safety (TeamSTEPPS) Teamwork Attitude Questionnaire (T-TAQ) [32]
- Team Skills Scale (TSS) [33]
- Student Perceptions of Interprofessional Clinical Education (SPICE-R2) [51]
- Healthcare Stereotypes Scale (HSS) [51]
- Interprofessional Socialization and Valuing Scale (ISVS) [52]

### Findings

**Fig. 2:**
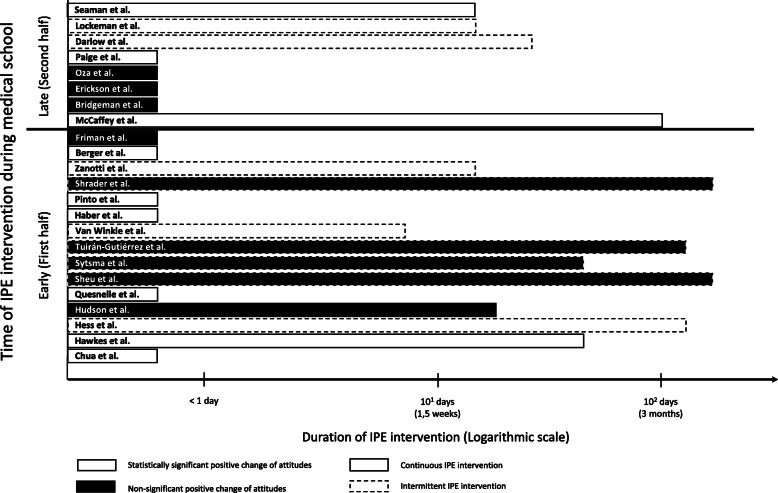
Bar chart: Outcome and duration of IPE interventions in selected articles, according to early (first half) or late (second half) time of medical school. White bars: statistically significant positive change of attitudes; Grey bars: Non-significant positive change of attitudes; full line: continuous IPE intervention; dotted line: intermittent IPE intervention

Over half of the studies (*n* = 13) [29, 32, 33, 36–39, 41, 43, 45, 49, 51, 52] showed a significant increase in positive attitudes towards IP after the interventions. Nine studies (39%) showed no significant changes in medical students’ attitudes towards IPE [30, 31, 40, 42, 44, 46–48, 50], while one demonstrated an increase in negative attitudes towards IPE after the intervention [10]. In years 1 and 2 IPE interventions appear longer in duration. Late IPE interventions show a trend to be longer and more statistically significant (Fig. 2). The sample size is too low for further comparisons.

### Methodological rigour

There was 91% agreement (kappa = 0.772) between the reviewers on the scores elicited by the McMaster Critical Review Form for Quantitative Studies [26], which represents good inter-rater reliability [54]. Consensus was reached on the disagreements after discussion. Methodological rigour scores ranged from 7 to 15 out of a maximum of 16. An additional word file shows the scoring in more detail [see Additional file 2]. Most studies (*n* = 18) were rated as either “Good” [10, 31, 36–38, 44, 47, 49, 51, 52], “Very Good” [29, 30, 39, 41, 45, 48] or “Excellent” [33].

### Meta-analysis

Initially we planned to undertake a meta-analysis of all studies included in the review. However, with such a broad range of instruments and therefore covering various different factors, it was not feasible. Instead, we performed the analysis with the RIPLS – as it was the most frequently used instrument –in the knowledge that this would only represent 26% of the articles in this review.

Due to the heterogeneity in the reporting of RIPLS results, a sound estimation of summary scores across studies was hampered. Whereas Darlow et al. [33] and Hudson et al. [10] used altered instruments with more than 19 items, Chua et al. [29], Paige et al. [32], Sheu et al. [30] and Sytsma et al. [31] used the original 19-item RIPLS. Nevertheless, in the article by Paige et al. [32], the item “*For small group learning to work, students need to trust and respect each other.”* is missing and the author did not respond to an email inquiring further information. Combined with extensive heterogeneity in reporting as well as statistically tested (Cochrane’s Q < 0.01 for the meta-analysis of Chua et al. [29], Paige et al. [32], Sheu et al. [30] and Sytsma et al. [31] for the subscales team, identity and role (see supplemental digital file Additional file 3/Table 3: Original RIPLS scores for Chua et al., Paige et al., Systma et al. and Sheu et al., supplemental_material_IPE_RIPLS_original_data.xls) the combination of the single study data for a summary measure seems prone to error. Additionally, authors used means and standard deviations in the original articles, which are not the appropriate summary measures for Likert scaled items. As Sheu et al. [30] only reported the means and standard deviations of RIPLS-subscales, a merging of information for meta-analysis was only possible on that level and not on a single item level. Furthermore, the standard deviations for the mean changes (difference of scores pre-test-post-test) were not given and had to be estimated according to Cochrane’s Handbook (16.1.3.2 Imputing standard deviations for changes from baseline), which introduced further uncertainty by the need to choose a rather random correlation coefficient of standard deviations (0.4 in our case). With regard to the pragmatic heterogeneity of interventions across studies, an ordinary pre-test-post-test score difference is a too simple way to capture the information created by the original studies. All in all, a meta-analysis could not be performed because of the high heterogeneity of the instruments used and the inconsistent data reporting.

## Discussion

In this systematic review, we analysed IPE interventions based on 23 studies published between 2011 and 2019. Our findings show that medical students were exposed to IPE interventions at various points in their training, and we could establish evidence of effectiveness of IPE. Three studies involved only medical students and therefore did not meet the WHO definition of IPE. However, they reported on interprofessional interventions and therefore were not excluded from this systematic review.

All years except the fifth study year were represented, so no preference for pre-clinical or clinical years could be observed. However, studies in the first four years of medical education were more frequent. This may reflect variation in the length of pre-registration medical education programmes worldwide. In the USA, medical school consists mainly of 4 years of training (generally preceded by a 3–4-year Bachelor’s degree), while in Europe it averages 6 years (without a preceding program) [55].

In Europe, most medical university programmes are public, and rather larger cohorts of students are educated (e.g., Germany has 36 public and only two private medical schools, and almost 10,000 new medical students per educational year, leading to an average class size of over 260 students) [56], while in the USA (141 fully-accredited medical schools), more than one third are private (*n* = 56) and class size is much smaller, with an average of 146 students per educational year [56, 57]. This may also explain the higher frequency of studies from the USA, as implementing IPE elements could be more feasible with smaller classes, and private medical schools may suffer more pressure to evaluate their programmes.

The optimal timing to introduce IPE is still subject to debate [10]. In clinical years it may seem reasonable, as it contributes to optimal development of students’ professional identities and gives them experience in working collaboratively with students in different health professions [11]. However, the introduction of IPE so late in the medical curriculum may be complicated by the students’ focus on profession-specific clinical practice [10]. On the other hand, introducing IPE early in pre-registration healthcare courses may be useful in breaking down negative attitudes and avoiding stereotypes [58–60].

From our analysis we could not determine the best time to introduce IPE, as both pre-clinical and clinical IPE interventions showed some degree of success. It appears that late IPE interventions show a trend to be longer and more statistically significant. It seems reasonable to conclude that interventions should be introduced in the early years and continue throughout the curriculum. More well-designed studies are needed to address this gap in knowledge.

Published IPE interventions had a pre-test-post-test design and most studies were cross-sectional. Interventions varied in their type and topic, group sizes were small and most activities were only performed once. There was also a paucity of studies reporting medium and long-term outcomes. Most studies (78%) were of good or very good quality, although a small proportion still scored poorly. This is consistent with previous reviews [4, 6, 15, 18]. This trend limits the development of strategies for targeting long-term behaviour changes and potential to positively impact patient outcomes. Longer interventions and longitudinal follow-up of learning outcomes are key to identifying robust outcomes that lead to changes in practice. An increasing number of studies now report mid- and long-term outcomes, but – as we can see from our own sample – these are still a minority. More studies are needed in models for pre-licensure IPE interventions (including adequate evaluation of their effectiveness), particularly regarding long-term outcomes [9, 31, 61]. In situations where prolonged IPE training is not feasible due to organizational limitations, intermittent interventions may be a good strategy [47]. The heterogeneity of most outcome measures may also limit the ability to draw conclusions about best practices and has, in our case, prevented the accomplishment of a meta-analysis.

Studies were most frequently assessed with RIPLS. The Readiness for Interprofessional Learning Scale, developed in 1999, was among the first scales developed for measurement of attitudes towards interprofessional learning [62]. It has been translated and acculturated into several languages [63]. The scale is very popular, but it has not been updated, it fails to embody all the dimensions of the Core Competencies for Interprofessional Collaborative Practice [2], and its conceptual framework has recently been questioned [63]. Additionally, concerns about its low internal consistency at item level and subscale results – raised by the RIPLS authors themselves – perpetuate the debate of what exactly the RIPLS is measuring [64] and there have even been past recommendations to abandon the scale altogether [23, 65]. Finally, some newer scales, more aligned with the IPEC dimensions, have also been successfully tested and acculturated [66, 67]. While educators, curriculum planners and policy makers continue to struggle to identify methods of interprofessional education that lead to better practice [9], clearer measures of interprofessional competency are needed to assess the outcomes from health professional degree programs and to determine what approaches to interprofessional education benefit patients and communities.

The results from this review and from individual studies should be interpreted with caution: students’ educational backgrounds, as well as attitudes, expectations and stereotypes, may vary considerably between institutions and countries and may influence how the IPE interventions are experienced. This probably accounts for many differences in effectiveness of IPE activities in different settings [15]. Additionally, a few studies described a “package” of interprofessional activities, and medical curricula differ significantly, which may introduce more bias. University IPE programmes should agree on a comparable methodology that aligns with research in IPE (e.g., larger cohorts, multi-centre studies) and should focus on fewer instruments to measure IPE, adequately assessed for validity, responsiveness, reliability, and interpretability [45].

There is a broad variation in the length of the medical curriculum between continents and countries. Most of the studies didn’t explain their specific curriculum to the reader. For many articles, we were not able to determine the total length of purported medical studies and therefore determine whether the IPE intervention took place in the final year, which would have been relevant to this literature review. To bridge this gap in knowledge we propose that future research should briefly describe their specific medical curriculum.

Our methodology also has limitations. We decided a priori to include only papers with a at least 35 medical students. The reason was to have sufficiently powered studies in the sample. However, this may have led to some selection bias, or left out potentially relevant interventions. Because we were interested in IPE effects on medical students, we also excluded all studies that did not report specific results for medical students. This limited the number of positive studies available. Similar to other systematic reviews, our work aimed to exclude all “lower quality” studies (i.e., non-randomised, non-experimental, qualitative studies) [9, 16, 20]. Reflecting on our methods, we question whether they are adequate for social or educational research, as there are repeated appeals for more qualitative reviews in IPE [61].

Unfortunately, there were also several issues that made a meta-analysis impossible. First, as RIPLS uses a Likert scale (therefore, an ordinal scale), central tendency statements should be calculated with the median value. However, most studies in this sample chose to report the mean. This is acceptable if one assumes equal distances between items, but it is very unrealistic. Additionally, students responding to pre- and post-intervention questionnaires were pooled cohorts, and items differed in wording (questionnaires were slightly modified). In given studies, some items were not reported. In other studies, items were sometimes scored reversely (negative attitudes), and some studies did not report the change in score which is the outcome of interest for the meta-analysis.

## Conclusions

This systematic review showed some evidence of a post-intervention change of attitudes towards IPE across different medical years studied. IPE was successfully introduced both in pre-clinical and clinical years of the medical curriculum. However, we found great variability in the scales chosen to evaluate changes in knowledge, behaviours and attitudes linked with participation in IPE. There was a paucity of studies reporting medium and long-term outcomes. The heterogeneity of results prevents further comparisons or the performance of a rigorous meta-analysis.

## Supplementary information

**Additional file 1.** Literature research for Review about interprofessional education for medical students. Detailed description of the search, including extracted hits, stratified by database.

**Additional file 2.** Methodological rigour assessment of the included studies using the modified McMaster Critical Review Form for Quantitative Studies.

**Additional file 3.** Table 3: Original RIPLS scores for Chua et al., Paige et al., Systma et al. and Sheu et al.

## Data Availability

All data generated and analysed during this study are included in this published article and its supplementary information files.

## Abbreviations

AHPQ: Attitudes to Health Professionals Questionnaire
ATCS: Attitudes Toward Collaboration Scale
ATHCTS: Attitudes Towards Health Care Teams Scale
ATITS: Attitudes Toward Interdisciplinary Teams Scale
CGI: Common Ground Instrument
HSS: Healthcare Stereotypes Scale
ICCAS: Interprofessional Collaborative Competency Attainment Scale
IEPS: Interdisciplinary Education Perception Scale
IPE: Interprofessional Education
IPEC: Interprofessional Education Collaborative
IPEC CSI: Interprofessional Educative Collaborative Competency Self-Assessment Instrument
ISVS: Interprofessional Socialization and Valuing Scale
JeffSPLL: Jefferson Scale of Physician Lifelong Learning
JSAPNC: Jefferson Scale of Attitudes toward Physician-Nurse Collaboration
JSE: Jefferson Scale of Empathy
PRISMA: Preferred Reporting Items for Systematic Reviews and Meta-analyses
PROSPERO: International prospective register of systematic reviews
RIPLS: Readiness for Interprofessional Learning Scale
SAMI: Sociocultural Attitudes in Medicine Inventory
SATP2C: Scale of Attitudes toward Physician-Pharmacist Collaboration
SEIEL: Self-Efficacy for Interprofessional Experimental Learning
SPICE-R2: Student Perceptions of Interprofessional Clinical Education
TAS: Teamwork Assessment Scale
TSS: Team Skills Scale
T-TAQ: Team Strategies and Tools to Enhance Performance and Patient Safety (TeamSTEPPS) Teamwork Attitude Questionnaire
UWE-IP-D: University of the West of England Interprofessional Questionnaire
