# Supplementary material for: Attitudes towards Interprofessional education in the medical curriculum: a systematic review of the literature
Source: BMC Med Educ. 2020 Aug 6;20:254. doi: 10.1186/s12909-020-02176-4 (PMC7410157; doi:10.1186/s12909-020-02176-4)
Supplement: Supplementary file 1 — Additional file 1. Literature research for Review about interprofessional education for medical students. Detailed description of the search, including extracted hits, stratified by database. [file 12909_2020_2176_MOESM1_ESM.docx]

**Literature research for Review about interprofessional education for medical students**

**Date of search 11-12-2019**

**Languages:**

English, German

**Idea:**

To comprehensively obtain all pubclications, which concern interprofessional education for medical students published after 2011 in English or German.

**Keywords:**

- “Interprofessional education” OR interprofession* OR "inter professional" OR "inter professionally" OR “ipe”
- "medical student" (terms for students excluded, because they yield a lot (hundreds to thousands) of publications concerning nursing students)

**Databases:**

Pubmed, PsycINFO, EThOS, EMBASE, PEDro, SCOPUS

**Pubmed :**

("interprofessional relations"[MeSH Terms] OR "interprofessional relations"[MeSH Terms] OR "interprofessional education"[ot] OR (interprofession[tiab] OR interprofessional[tiab] OR interprofessional'[tiab] OR interprofessionalality[tiab] OR interprofessionalcommunity[tiab] OR interprofessionalism[tiab] OR interprofessionality[tiab] OR interprofessionally[tiab] OR interprofessionals[tiab] OR interprofessionals'[tiab] OR interprofessionel[tiab] OR interprofessionell[tiab] OR interprofessionelle[tiab] OR interprofessionelles[tiab] OR interprofessionlity[tiab] OR interprofessionnal[tiab] OR interprofessionnalite[tiab] OR interprofessionnality[tiab] OR interprofessionnel[tiab] OR interprofessionnelle[tiab] OR interprofessionnelles[tiab] OR interprofessionnels[tiab]) OR "inter professional"[tiab] OR "inter professionally"[tiab] OR "ipe"[ot]) AND ("students, medical"[MeSH Terms] OR "medical student"[ot]) AND ("2011/01/01"[PDAT] : "2019/12/11"[PDAT])

Hits: 645

Endnote-library with 3 new hits since 26-09-2019, but difference in total items is 26. Most likely, because articles published before 26^th^ of September, but only indexed after the 26^th^ of September are now included…

**SCOPUS:**

TITLE-ABS-KEY(("interprofessional education" OR "inter professional" OR "ipe") AND ("medical student" OR "student")) AND PUBYEAR > 2010 AND ( LIMIT-TO ( LANGUAGE,"English" ) OR LIMIT-TO ( LANGUAGE,"German" ) )

Hits: 2004 Hits only 2019/2020: 332

Comment: Only Title, Abstract and Keywords searched, otherwise more than 7000 hits.

**EMBASE:**

((Interprofessional education or interprofession$ or inter professional or inter professionally or ipe) and medical student).af.

limit 1 to yr="2011 -Current"

Hits: 955 Hits only 2019/2020: 120

Comment: Only possible to limit language option to a single language, therefore no limitations. Results will include a few articles in languages other than English and German.

**PsychINFO:**

((interprofessional education or interprofession* or inter professional or inter professionally or ipe) and medical student).af.

limit 2 to yr="2011 -Current"

Hits: 358 Hits only 2019: 18 We have less hits (around 120) then in September and I do not see why. It’s very unlikely we would include one of the hits from the search in September and miss it with this search…

Comment: Only possible to limit language option to a single language, therefore no limitations. Results will include a few articles in languages other than English and German.

**PEDro (simple search):**

“interprofessional education”

Hits: 5 – none is relevant

**EthOS:**

interprofessional education AND medical student

Hits: 7 – thesis, no journal articles, so no export to EndNote.

All hits: 3974
