# Supplementary material for: Attitudes towards Interprofessional education in the medical curriculum: a systematic review of the literature
Source: BMC Med Educ. 2020 Aug 6;20:254. doi: 10.1186/s12909-020-02176-4 (PMC7410157; doi:10.1186/s12909-020-02176-4)
Supplement: Supplementary file 2 — Additional file 2. Methodological rigour assessment of the included studies using the modified McMaster Critical Review Form for Quantitative Studies. [file 12909_2020_2176_MOESM2_ESM.docx]

| Criterion | Berger et al.[41] | Bridgeman et al.[42] | Chua et al. [28] | Darlow et al. [47] | Erickson et al.[44] | Friman et al.[43] | Haber et al. [36] | Hawkes et al. [29] | Hess et al. [30] | Hudson et al.[11] | Lockeman et al.[48] |
| --- | --- | --- | --- | --- | --- | --- | --- | --- | --- | --- | --- |
| 1. Study purpose clearly stated | 1 | 1 | 1 | 1 | 1 | 1 | 1 | 1 | 1 | 1 | 1 |
| 1. Literature review relevant | 1 | 1 | 1 | 1 | 1 | 1 | 1 | 1 | 1 | 1 | 1 |
| 1. Study design appropriate to study aims | 1 | 1 | 1 | 1 | 1 | 1 | 1 | 1 | 1 | 1 | 1 |
| 1. No biases present | 0 | 0 | 0 | 0 | 0 | 0 | 0 | 0 | 0 | 0 | 0 |
| 1. Sample described in detail | 0 | 0 | 1 | 1 | 1 | 1 | 0 | 0 | 0 | 0 | 1 |
| 1. Sample size justified | 0 | 0 | 0 | 1 | 0 | 0 | 0 | 0 | 0 | 0 | 0 |
| 1. Informed consent gained | 0 | 0 | 1 | 1 | 0 | 1 | 1 | 0 | 0 | 0 | 0 |
| 1. Validity of outcome measures used | 0 | 1 | 1 | 1 | 0 | 1 | 1 | 1 | 1 | 1 | 0 |
| 1. Reliability of outcome measures used | 0 | 1 | 1 | 1 | 0 | 1 | 1 | 1 | 1 | 1 | 1 |
| 1. Intervention described in detail | 1 | 1 | 1 | 1 | 1 | 1 | 1 | 0 | 1 | 1 | 1 |
| 1. Statistical reporting of results | 1 | 1 | 1 | 1 | 1 | 1 | 1 | 1 | 1 | 1 | 1 |
| 1. Appropriate statistical analysis | 0 | 1 | 1 | 1 | 1 | 1 | 1 | 1 | 1 | 1 | 1 |
| 1. Clinical importance reported | 1 | 1 | 1 | 1 | 1 | 1 | 1 | 1 | 1 | 1 | 1 |
| 1. Appropriate conclusions | 1 | 1 | 1 | 1 | 1 | 1 | 1 | 1 | 1 | 1 | 1 |
| 1. Clinical implications reported | 1 | 1 | 1 | 1 | 1 | 1 | 1 | 1 | 1 | 1 | 1 |
| 1. Study limitations acknowledged | 1 | 1 | 1 | 1 | 1 | 1 | 1 | 1 | 1 | 1 | 1 |
| Total | 9 | 12 | 14 | 15 | 11 | 14 | 13 | 11 | 12 | 12 | 12 |
| Descriptor | Fair | Good | Very Good | Excellent | Good | Very Good | Very Good | Good | Good | Good | Good |

**Table 3: Methodological rigour assessment of the included studies using the modified McMaster Critical Review Form for Quantitative Studies[25]**

**Table 3 (cont.): Methodological rigour assessment of the included studies using the modified McMaster Critical Review Form for Quantitative Studies[25]**

| Criterion | McCaffrey et al. [37] | Oza et al. [45] | Paige et al. [46] | Pinto et al. [38] | Quesnelle et al. [31] | Seaman et al. [49] | Sheu et al. [32] | Shrader et al. [39] | Sytsma et al.[33] | Tuiran-guiterrez et al. [34] | Van Winkle et al. [35] | Zanotti et al.[40] |
| --- | --- | --- | --- | --- | --- | --- | --- | --- | --- | --- | --- | --- |
| 1. Study purpose clearly stated | 1 | 1 | 1 | 1 | 1 | 1 | 1 | 1 | 1 | 1 | 1 | 1 |
| 2. Literature review relevant | 1 | 1 | 1 | 1 | 1 | 1 | 1 | 1 | 1 | 1 | 1 | 1 |
| 3. Study design appropriate to study aims | 1 | 1 | 1 | 1 | 1 | 1 | 1 | 1 | 1 | 1 | 1 | 1 |
| 4. No biases present | 0 | 0 | 0 | 0 | 0 | 0 | 0 | 0 | 0 | 0 | 0 | 0 |
| 5. Sample described in detail | 0 | 0 | 0 | 0 | 0 | 1 | 1 | 0 | 0 | 1 | 0 | 1 |
| 6. Sample size justified | 0 | 0 | 0 | 0 | 0 | 0 | 0 | 0 | 0 | 0 | 0 | 0 |
| 7. Informed consent gained | 0 | 0 | 0 | 0 | 0 | 1 | 0 | 0 | 0 | 1 | 0 | 0 |
| 8. Validity of outcome measures used | 0 | 0 | 0 | 0 | 1 | 0 | 1 | 1 | 1 | 1 | 1 | 1 |
| 9. Reliability of outcome measures used | 0 | 0 | 0 | 0 | 1 | 0 | 1 | 1 | 1 | 1 | 1 | 1 |
| 10. Intervention described in detail | 0 | 1 | 1 | 0 | 1 | 0 | 1 | 1 | 1 | 1 | 1 | 1 |
| 11. Statistical reporting of results | 0 | 1 | 1 | 1 | 1 | 1 | 1 | 1 | 1 | 1 | 1 | 1 |
| 12. Appropriate statistical analysis | 0 | 1 | 1 | 1 | 1 | 1 | 1 | 1 | 1 | 1 | 1 | 1 |
| 13. Clinical importance reported | 1 | 1 | 1 | 1 | 1 | 1 | 1 | 1 | 1 | 1 | 1 | 1 |
| 14. Appropriate conclusions | 1 | 1 | 1 | 1 | 1 | 1 | 1 | 1 | 1 | 1 | 1 | 1 |
| 15. Clinical implications reported | 1 | 1 | 1 | 1 | 1 | 1 | 1 | 1 | 1 | 1 | 1 | 1 |
| 16. Study limitations acknowledged | 1 | 1 | 1 | 1 | 0 | 1 | 1 | 1 | 1 | 0 | 1 | 1 |
| Total | 7 | 10 | 10 | 9 | 11 | 11 | 13 | 12 | 12 | 13 | 12 | 13 |
| Descriptor | Poor | Fair | Fair | Fair | Good | Good | Very Good | Good | Good | Very Good | Good | Very Good |
